# Arrhythmias across the tree of life: comparative insights for human electrophysiology

**DOI:** 10.3389/fcvm.2025.1652591

**Published:** 2026-01-06

**Authors:** Barbara Natterson-Horowitz, Kathy Wright, Glenn Van Steenkiste, Annelies Decloedt, Allison Lynne Gagnon, Xinjiang Cai, Alin Mazmanian

**Affiliations:** 1 Division of Cardiology, David Geffen School of Medicine at UCLA, Los Angeles, CA, United States; 2 Department of Global Health and Social Medicine, Harvard Medical School, Boston, MA, United States; 3 Department of Cardiology, MedVet Medical & Cancer Centers for Pets, Fairfax, OH, United States; 4 Equine Cardioteam Ghent, Department of Internal Medicine, Reproduction and Population Medicine, Ghent University, Ghent, Belgium; 5 Department of Medicine and Epidemiology, School of Veterinary Medicine at University of California-Davis, Davis, CA, United States; 6 Department of Ecology and Evolutionary Biology, University of California, Los Angeles, CA, United States

**Keywords:** comparative electrophysiology, atrial fibrillation, ventricular tachycardia, veterinary cardiology, translational medicine, breed-specific arrhythmias, sudden cardiac death, cross-species thromboresistance

## Abstract

**Introduction:**

Arrhythmias in non-human animals offer insights into human electrophysiology, yet physicians may be unaware of their occurrence and significance. This paper presents selected examples of arrhythmias in dogs, horses, and birds— as an invitation to human cardiologists to explore how animal models can illuminate mechanisms, genetics, and therapeutic approaches relevant to human electrophysiology.

**Methods:**

Leading veterinary cardiologists compiled overviews of common arrhythmias in dogs, cats, horses and birds. Genetic predisposition, natural history, therapeutic approaches, and epidemiology were compared across these species and humans, highlighting translational opportunities.

**Results:**

Common human arrhythmias including atrial fibrillation, bradycardia, ventricular tachycardia, and arrhythmogenic right ventricular cardiomyopathy occur naturally in dogs, cats, horses, and birds. Cross-species differences in disease expression provide unique insights into mechanisms of arrhythmia vulnerability and resistance. Dogs develop similar inherited arrhythmogenic diseases but with distinct phenotypes. Horses experience atrial fibrillation without thromboembolic complications, revealing potential protective pathways. They also demonstrate extreme exercise-induced arrhythmia susceptibility, isolating exercise as an arrhythmogenic trigger. Avian species exhibit remarkable adaptation to cardiac loading conditions that would be pathological in mammals. These comparative observations across species highlight novel mechanisms underlying both susceptibility and resistance to arrhythmias and conduction disorders, offering unexplored therapeutic targets for human patients.

**Discussion:**

Cross-species knowledge offers direct translational value for human electrophysiology—from genetic markers in Labrador Retrievers with supraventricular tachycardia to cardiac loading paradigms in broiler chickens. Breaking down disciplinary barriers through shared research initiatives and integrated training represents an essential, underutilized strategy for advancing arrhythmia diagnosis, treatment, and prevention in human patients.

## 1 Introduction

**Figure 1:**
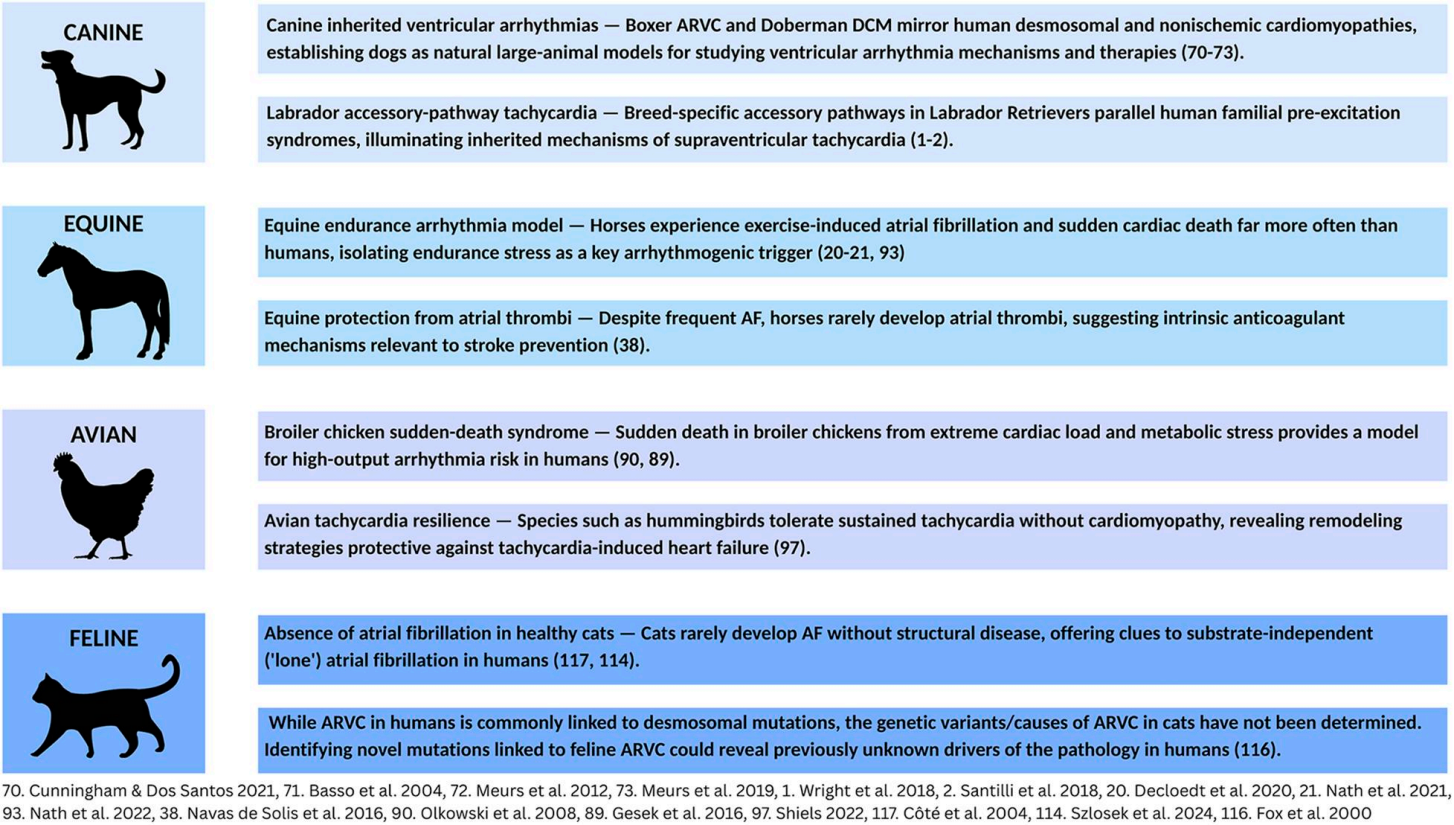
Arrhythmias in animals: a source of translational insights for human EP. Common clinical arrhythmias in dogs, horses, birds, and cats offer unique insights into challenges in human electrophysiology.

Many human electrophysiologic disorders also occur in other species. Veterinary cardiologists have extensive experience diagnosing, managing, and preventing these pathologies. Their insights can strengthen our understanding of human arrhythmias. Unfortunately, human cardiovascular training rarely includes exposure to these veterinary challenges. This paper seeks to bridge this gap by presenting a collection of clinically significant arrhythmias in dogs, horses, and birds along with human translational insights emerging from this comparative knowledge (Figure 1).

Most scientific literature on animal arrhythmias focuses on taxa under human care and oversight, although arrhythmia and conduction abnormalities exist across all vertebrate taxa. Much of the veterinary literature is focused on companion animals with breed-specific risks for electrophysiologic disorders, equine athletes, particularly Standardbred and Thoroughbred horses and agricultural birds (1–3).

## 2 Atrial fibrillation

### 2.1 AF in dogs

Atrial fibrillation (AF) is the most common canine arrhythmia, diagnosed in 6.3–10.5% of dogs presenting with clinical heart disease and representing approximately one third of all pathologic arrhythmias (4, 5). Genetic factors and breed type play significant roles in susceptibility to AF (6). In Irish Wolfhounds, for example, AF incidence may exceed 10% while likelihood of AF in Miniature Poodles is as low as.04% (7, 8). Breed and size are linked, a factor influencing the nearly 6-fold increased risk of developing AF in large dogs (>20 kg) vs. smaller individuals (9). Atrial enlargement, left ventricular dimension and body weight are major risk factors associated with risk of developing AF (10–12).

In dogs, AF develops most often in association with structural heart disease (SHD). Dilated cardiomyopathy (DCM) and advanced myxomatous mitral valve degeneration (MMVD) are the two most common underlying structural abnormalities linked to AF (13, 14). MMVD more commonly affects smaller dogs and DCM most commonly occurs in larger dogs. Primary AF (AF in the absence of SHD) is generally diagnosed in giant breed dogs. Since multiple reentrant circuits are needed to maintain AF, it may be that larger breeds are at increased risk compared to smaller breeds because they have sufficiently large atrial surfaces to support these electrophysiologic mechanisms (9). Age and sex may be predictors of AF in dogs; however, the strength of these associations has recently been challenged (12, 15). Severe MMVD commonly affects small to medium size dogs; however, even within the MMVD population, larger breed dogs with advanced disease and concurrent congestive heart failure (CHF) are especially at risk of developing AF (14, 16). Dogs with accessory atrioventricular pathways (AVPs) are predisposed to AF. In some cases, ablation of the AVP can eliminate or markedly reduce AF (1, 17). The goal of pharmacological treatment of AF in dogs is not cardioversion to sinus rhythm but maintenance of a ventricular rate no more than 120–125 bpm (18, 19).

### 2.2 AF in horses

In horses, AF prevalence ranges from 0.3% to 2.5% (20), reaching 4.9% in thoroughbreds (21). In equine athletes, AF is the most common arrhythmia causing poor performance (22), with racehorses and other athletes at higher risk due to intense cardiac demands (23). Large breed horses are more susceptible because greater atrial size may provide the critical mass needed to sustain arrhythmia (20, 24), which may explain its rarity in smaller horses, ponies, and foals (25).

SHD, including congenital abnormalities and valvular insufficiencies, can predispose horses to AF (26, 27), however, most cases occur in apparently normal hearts. This condition may be triggered by vigorous exercise, electrolyte imbalance, or genetic predisposition. Sustained AF (48 h or more is considered persistent) may be treated with pharmacological or electrical cardioversion. Success depends on specific characteristics of the horse and the AF itself (28). Many horses can be successfully converted to normal rhythm, though some remain in permanent AF.

### 2.3 AF in birds

The translational insights for human AF from avian species is less straightforward than in dogs and horses. A significant difference is that unlike mammals, birds have a single pulmonary vein entering the left atrium. They may therefore lack the complex, arrhythmogenic pulmonary venous anatomy that often serves as the origin of AF in humans and other mammals (29).

### 2.4 Comparison of AF to humans

In both humans and dogs, AF is strongly linked to underlying SHD such as left atrial enlargement (10–12, 30). Larger overall body size also appears to increase risk in humans and dogs (11, 12). While age is a strong predictor of AF in humans, a similar association in dogs is not definitively established (11, 12, 15, 31). The natural history of AF in dogs and humans also differs with respect to thrombogenicity. A major risk associated with AF in humans is left atrial thrombosis and thromboembolic events (32, 33). By contrast, AF in dogs, even with left atrial enlargement, is rarely not associated with thrombosis or embolic events (34–36). Differences in atrial remodeling between the species with AF may underlie this variation between species (37).

A similar “resistance” to left atrial thrombus with AF is also found in equine patients. In horses, even longstanding AF does not appear to increase the risk of left atrial thrombus or clinical embolic events (38). Equine athletes also provide valuable comparative insights into AF. In both humans and horses, endurance and high-intensity athletes have a higher incidence of the arrhythmia. This points to a shared, atrial stress mechanism during strenuous exercise that may underlie AF initiation. A genetic component is present in both, and breed-linked AF in horses offers insights into specific genetic pathways relevant to human AF (39, 40). Approach to treatment is similar between the species, including the use of both pharmacological or electrical cardioversion when warranted (28).

## 3 Supraventricular tachycardia

### 3.1 SVT in dogs

Supraventricular tachycardia (SVT) secondary to accessory pathways (APs) has been identified in at least 37 dog breeds. Males account for about two-thirds of all cases (1, 2). Labrador Retrievers are especially vulnerable, comprising nearly half of North American and over a third of European dogs with confirmed APs, suggesting a strong genetic risk (1, 2). While breed predispositions exist for APs, none have been identified for focal atrial tachycardias (FATs) (41). Canine APs at electrophysiologic study (EPS) have unique features, with about 93% of canine APs located on the tricuspid annulus - a significant difference to humans, where they are most often on the mitral annulus (1, 2, 41). FATs, by contrast, originate from a single point in the atria (42).

Definitive diagnosis of SVT in dogs requires EPS under general anesthesia, making some types of SVT harder to detect (43). Once diagnosed, radiofrequency catheter ablation (RFCA), a procedure that uses heat to destroy the abnormal electrical tissue, is a common and effective treatment (1).

### 3.2 SVT in horses

Atrial tachycardia (AT) is the most common SVT in horses. One common mechanism is a macroreentrant circuit near the myocardial sleeves of the caudal vena cava (44), although FATs may also originate from a single atrial focus. Although “AT” is often used as a general term encompassing both focal and reentrant atrial tachycardias, it is important to distinguish these from other types of SVT such as atrioventricular reentrant tachycardia (AVRT) (45). Most cases are diagnosed in sport or performance horses undergoing cardiac evaluation. Recent advances, such as three-dimensional electro-anatomical mapping, have allowed precise localization of arrhythmia electrical activity. A consequence has been successful treatment of macroreentrant AT and AVPs (46–48). RFCA can eliminate the abnormal tissue and holds promise as a definitive therapy, despite the horse's large size and thick atrial walls (10–20 mm in the left atrium) (47).

### 3.3 SVT in birds

SVT has been diagnosed in birds in a variety of clinical contexts (49–51). Abnormal heart development have been documented to lead to the persistence of APs in a chick embryo model (52). Infection with avian influenza virus has been linked to AT in chickens and supraventricular premature complexes in other species (49). Arrhythmia-induced cardiomyopathy is also diagnosed in birds; clinical presentation includes left ventricular enlargement and poor cardiac function (51).

### 3.4 Comparison of SVT to humans

In both humans and dogs, APs and reentrant circuits are common mechanisms linked to SVT (53–56). Differences can be found in the location of the AP; in dogs 93% occur along the tricuspid annulus (1, 2, 41), while in humans 50%–60% are along the mitral annulus. Ventricular preexcitation linked to Wolff-Parkinson-White in humans is significantly less common in dogs (57). Genetics plays a central role in vulnerability. Specific breeds are at elevated risk. For example, Labrador Retrievers comprise up to 46% of dogs (North American study) with confirmed APs (1). This parallels the central role of genetics in human SVTs; ion channel gene variants are commonly implicated in our species (58).

Both human and equine athletes who participate in intense exercise may develop changes known as “athlete's heart” (59, 60). Athletes of both species are at increased risk of SVT; however, the specific causes and the anatomical locations of the arrhythmias differ significantly between the two species. In human athletes, SVT often arises from an AP (like in Wolff-Parkinson-White syndrome) or a reentrant circuit within the AV node (AVNRT), usually located in the left atrium (61). Sympathetic activation and higher heart rates enhance accessory conduction and facilitate reentry. In contrast, SVT in horses is more frequently associated with macroreentrant circuits (44), commonly involving the caudal vena cava where myocardial sleeves can become a site for arrhythmogenesis, creating a reentrant circuit. This AT mechanism is rarely documented in humans.

In both humans and birds, sustained SVT may lead to cardiomyocyte damage and arrhythmia-induced cardiomyopathy where a persistent tachycardia weakens the heart muscle over time (52). While APs are a common cause of SVT in humans, the persistence of these pathways in birds is linked to normal developmental processes (49, 52, 61, 62). Viral infection has been linked to SVT in avian species, but is not a typical cause in humans (49).

## 4 Ventricular tachycardia

### 4.1 VT in dogs

Ventricular tachycardia (VT) in dogs is often due to inherited heart diseases (63–68). Primary causes and characteristics of VT vary across vulnerable breeds including Boxers, Doberman Pinschers, and German Shepherds (GSD) (69).

Arrhythmogenic Right Ventricular Cardiomyopathy (ARVC) occurs most commonly in Boxers and English Bulldogs, though other breeds may also be affected (70). It is associated with sudden cardiac death and high cardiovascular morbidity (71) with average age (6 years) at presentation in Boxers (70). VT is a leading cause of sudden death in Doberman Pinschers with inherited DCM (72, 73). Females have increased risk of VT, while males tend to show earlier echocardiographic changes (74, 75). Rapid, sustained VT may require intravenous antiarrhythmic drugs or emergent direct current cardioversion. RFCA has been successfully performed in limited cases (76, 77).

A juvenile form of VT affects primarily GSDs although it occurs less commonly in Rhodesian Ridgebacks, Leonbergers and other breeds (63, 78, 79). Affected GSDs develop polymorphic ventricular arrhythmias (VAs) around 12 weeks of age. Sudden cardiac death (SCD) may be the presenting event (63). These ventricular arrhythmias originate from triggered activity in the left ventricular Purkinje fibers (80) (not related to QT prolongation) and resolve spontaneously if the dog survives past two years of age (69). Treatment includes the use of antiarrhythmic drugs.

### 4.2 VT in horses

VAs are relatively common in horses, particularly athletes. Several studies report a high prevalence of premature ventricular complexes (PVCs) in clinically healthy, well-performing sport horses (81–83). VT is a serious condition and may cause poor performance, weakness, collapse or SCD (84). Horses at highest risk are athletes, racehorses and sport horses, experiencing significant cardiac stress while undergoing intense training (84). VT often emerges during or immediately following strenuous exercise, although it is sometimes detected at rest (3).

Although the cause of VT in horses often cannot be determined, it can be seen in association with underlying cardiac disorders such as myocarditis or systemic illness, colic and electrolyte imbalance (85). These conditions may promote electrical instability in the ventricles, predisposing the patient to VT. When life-threatening or symptomatic, treatment with antiarrhythmic drugs is required (85).

### 4.3 VT in birds

VAs are most frequently identified in avian species that maintain high basal heart rates and experience extreme cardiac loading due to a high metabolic rate due to rapid growth and/or acute physiological stress (86). Broilers have been bred to develop significant muscle mass (87). For broiler chickens in the rapid growth phase, high cardiac demand may lead to acute heart failure, with VAs and SCD being a risk in this setting (88, 89). VAs may emerge in the setting of systemic illnesses, exposure to toxins such as ingestion of heavy metals, or as a consequence of primary myocardial diseases such as myocarditis, or cardiomyopathy (62, 90).

### 4.4 Comparison of VT to humans

VT in dogs offers a naturally occurring large-animal model with significant translational value for human electrophysiology. Canine diseases closely mirror human conditions: Boxers develop VT associated with ARVC while Dobermans show VT linked to DCM, directly modeling human ARVC and DCM natural history and structural characteristics (69, 72, 73). Juvenile VT in GSDs exhibit a rare phenotype that may help electrophysiologists understand non-structural, triggered activity-based mechanisms in humans (91). This juvenile arrhythmia is a self-resolving, pause-dependent polymorphic VT (63), likely originating from left ventricular Purkinje fibers rather than scar tissue. This model is valuable for testing Purkinje-targeted interventions and improving risk stratification in genetically predisposed human populations.

Horses provide distinct comparative models for human VT and SCD. Cardiovascular causes are presumed when necropsy reveals no other explanation for equine sudden death (92). Unlike humans, horses rarely develop inherited cardiomyopathies – perhaps due to performance selection – yet, exercise-related SCD occurs 200 times more often in horses than humans (93). Racing's extreme physiologic demand creates challenging electrical environments: heart rate ranges from 28 bpm at rest to 240 bpm maximally, causing heterogeneous refractory dispersal post-exercise (3). While human VT usually arises from coronary disease and post-infarction scarring establishing re-entrant circuits, horses rarely develop coronary disease; their VT links to intense training, AF, systemic illness, or electrolyte disturbances (84, 85, 94, 95). Clinically, equine VT may manifest as poor performance, whereas humans most often experience syncope or cardiac arrest (84, 96).

Studying exercise-related arrhythmias in horses, including possible ion channelopathies, offers insights into repolarization instability elevating SCD risk in human athletes. Translational insights come from SCD in broiler chickens with load-associated pathology. Rapid growth creates high cardiac output demands and excessive cardiac afterload causing left ventricular dysfunction and heart failure (86). Avian SCD provides a natural model relevant for ventricular arrhythmias in high-load human cardiomyopathy (86). Birds may also model tachycardia-induced cardiomyopathy resistance; hummingbirds sustain flight heart rates over 600 bpm without the cardiomyopathic changes seen in humans (97, 98). Examining avian susceptibility and resistance to arrhythmias may provide novel insights for preventing and managing cardiomyopathy and sudden death risk in vulnerable human populations.

## 5 Bradyarrhythmias

### 5.1 Bradyarrhythmias in dogs

The most common bradyarrhythmia in dogs is sinus arrhythmia, a non-pathological condition. Sick sinus syndrome (SSS) and high-grade atrioventricular (AV) blocks are the most clinically significant bradyarrhythmias (99, 100). These typically are NOT linked to underlying SHD, but are degenerative processes that have breed predilections. High vagal tone in resting or sedated dogs can accentuate the underlying EP abnormality (100, 101). Genetics affects susceptibility; Miniature Schnauzers and Cocker Spaniels more commonly present with SSS, while larger breeds are prone to AV block (102, 103). Symptomatic dogs present with lethargy, exercise intolerance, weakness, and syncope, with severe cases exhibiting sudden death (104, 105).

### 5.2 Bradyarrhythmias in horses

The most frequent bradyarrhythmias in horses are second-degree AV block and sinus bradycardia (106, 107). Second-degree AV block can be normal in well-conditioned athletes (108), but can also indicate myocarditis, electrolyte disturbances, or systemic illnesses (107, 109–112). In equine athletes, second-degree AV block is often asymptomatic (106) although advanced second-degree AV block may result in poor athletic performance, exercise intolerance, or collapse (107).

### 5.3 Comparison of bradyarrhythmias to humans

Dogs and humans have similar sinoatrial node structure and function. In both species, SSS is a common clinical indication for pacemaker treatment (113). Vulnerable breeds like Miniature Schnauzers offer a valuable model for understanding genetic bases and mechanisms of SSS. Developing therapies and pacemaker technology for smaller dogs may directly inform strategies for treating bradyarrhythmias for smaller adults and pediatric patients.

In equine athletes, bradycardias - especially second-degree AV block - may be normal, related to high vagal tone and cardiac efficiency (106, 108). Equine cardiac function across extreme heart rates can strengthen our ability to distinguish benign from pathological second-degree AV block and other bradyarrhythmias in athletic humans.

## 6 The feline model

Arrhythmias are less common in cats than dogs, typically occurring with systemic illness or underlying SHD in both species. The most frequent feline arrhythmias in a recent 9,000+ cat study were PVCs, though cats also presented with premature atrial contractions (PACs), SVT, AT, AF and AV blocks (114).

Ventricular arrhythmias emerge primarily in the setting of systemic illness or underlying SHD. In cats, hypertrophic cardiomyopathy (HCM) is the most common serious cardiovascular disease (115). In HCM, myofibrillar disarray disrupts conduction tissue causing arrhythmias. ARVC occurs far less commonly than HCM; however, fatty and fibrous replacement of normal tissue, especially in the right ventricle, may be arrhythmogenic (116).

Unlike dogs, cats rarely develop arrhythmias in structurally normal hearts. Their smaller cardiac dimensions—especially a normal left atrial diameter of roughly 12 mm—limit the atrial surface area required to sustain reentrant arrhythmias such as AF (117). This anatomic constraint parallels observations in small and medium-sized dogs, which also do not develop AF without marked atrial dilation.

AF, when it does occur, is therefore almost always secondary to advanced SHD with significant atrial enlargement (117, 118). Importantly, atrial dilation in cats carries a high risk of left atrial thrombus formation and systemic thromboembolism, including aortic “saddle” thrombus, which can cause acute limb ischemia. This propensity contrasts sharply with the relative thromboresistance seen in dogs and horses with AF, and more closely resembles human AF pathophysiology.

The heightened feline thrombotic risk likely reflects species-specific differences in coagulation and endocardial response to stasis, as well as the compact geometry of the feline left atrium, which promotes blood stasis once dilation occurs (117; 38). Recognizing this distinction provides a valuable comparative model for understanding atrial thrombogenesis in humans.

### 6.1 Comparison of feline arrhythmias to humans

Cats, unlike humans, rarely develop AF without severe underlying heart disease (118). This may provide insight into lone AF vulnerability in humans. Moreover, while feline AF almost invariably leads to thromboembolism, human and feline AF share similar hemodynamic and prothrombotic mechanisms, making cats an important natural model for AF-associated stroke risk. Differences between human and feline ARVC and HCM—shared sarcomeric mutations in HCM but absent desmosomal mutations in feline ARVC—offer valuable insights for human patients (119, 120).

## 7 Conclusion

A comparative survey of arrhythmia across species reveals potential models for human electrophysiology. Examples with translational potential for humans include resistance to AF-associated thromboembolic events in horses and dogs, canine breed-specific arrhythmia predispositions, and a sudden death syndrome linked to acute heart failure in broiler chickens. In contrast, cats provide a unique natural model of thromboembolic vulnerability, highlighting how small atrial size and species-specific coagulation profiles can amplify embolic risk once AF develops—a finding that strengthens the translational value of feline cardiomyopathy research for human stroke prevention.
